# Screening of early predictive serum biomarkers and construction of a combined predictive model for refractory *Mycoplasma pneumoniae* infection in children

**DOI:** 10.3389/fped.2026.1680913

**Published:** 2026-02-19

**Authors:** Ling Zhu, Jinsheng Xu, Tailei Yuan, Haoyue Li, Jun Li

**Affiliations:** 1 Pediatrics Unit, Nanjing Jiangbei Hospital, Nanjing, China; 2 Clinical Laboratory, Nanjing Jiangbei Hospital, Nanjing, China; 3 Clinical Medicine, Clinical Medical College of Anhui Medical University, Hefei, China

**Keywords:** *mycoplasma*, *pneumonia*, predictive model, respiratory infection, serum biomarkers

## Abstract

**Background:**

*Mycoplasma pneumoniae* pneumonia (MPP) is a prevalent respiratory infection. Refractory MPP (RMPP) presents more severe symptoms and requires more intensive treatment compared to general MPP (GMPP). This study aimed to identify distinguishing clinical, laboratory, and radiological characteristics between RMPP and GMPP and develop an early predictive model for RMPP risk stratification.

**Methods:**

A total of 568 patients, including 130 RMPP cases and 438 GMPP cases, were enrolled. Clinical information, laboratory tests, and radiological features were compared. Univariate and multivariate logistic regression analyses identified serum biomarkers associated with RMPP. A combined predictive model using random forest approach was developed and externally validated.

**Results:**

RMPP patients showed significantly higher rates of older age, fever, tachypnea, chest tightness, wheezing, chills, extrapulmonary complications, decreased unilateral lung sounds, longer fever duration, hospital stay, antibiotic therapy, oxygenotherapy use, and Intensive Care Unit (ICU) admission (all *P* < 0.05). Laboratory findings revealed elevated neutrophil percentage, C-reactive protein (CRP), lactate dehydrogenase (LDH), immunoglobulin A (IgA), interleukin (IL)-6, IL-10, and interferon-gamma (IFN-γ), but lower prealbumin (PAB) concentrations in RMPP. Radiologically, RMPP exhibited more severe manifestations such as large lesions, pleural effusion, lobar atelectasis, pulmonary consolidation, and pleural thickening. Using the eight independently associated serum biomarkers, we developed a multi-factor random forest model that showed excellent discrimination between RMPP and GMPP (AUC = 0.978 in the development cohort), which was confirmed in an external validation cohort (AUC = 0.957).

**Conclusions:**

Significant differences in clinical, laboratory, and radiological characteristics were observed between RMPP and GMPP. The combined multi-marker model shows strong potential for early risk identification of RMPP and may support timely clinical decision-making; however, prospective validation is needed before routine implementation.

## 1 Introduction

*Mycoplasma pneumoniae* (MP) is one of the primary pathogens causing community-acquired pneumonia (CAP) in children, accounting for 10%–40% of cases, with an infection rate as high as 50% in children over five years old (1, 2). In recent years, several regions have reported a rising proportion of refractory or severe MP infections, reflecting both increasing clinical burden and greater recognition of treatment-resistant disease. Although most patients with *Mycoplasma pneumoniae* pneumonia (MPP) respond well to macrolide antibiotic treatment, some develop refractory *Mycoplasma pneumoniae* pneumonia (RMPP), characterized by persistent fever, radiological progression, and multi-organ complications, which can be life-threatening (3). RMPP has a prolonged course, and conventional anti-infective treatments are often ineffective. If not identified early and treated appropriately, RMPP can progress to necrotizing pneumonia, resulting in pleural effusion, respiratory distress, septic shock, pulmonary embolism, Mycoplasma encephalitis, and liver dysfunction, severely impacting the child's quality of life and potentially being fatal. Later complications include bronchiolitis obliterans, bronchiectasis, and interstitial lung disease, leading to poor exercise tolerance (4, 5).

An RMPP risk prediction model can help clinicians identify high-risk children early and provide timely appropriate treatments, significantly reducing complications, sequelae, and improving clinical outcomes (6, 7). The clinical features of RMPP are complex, involving mechanisms such as host immune response imbalance, excessive activation of inflammatory factors, and direct lung injury. In addition, increasing macrolide-resistant MP strains commonly associated with 23S rRNA mutations have been recognized as an important contributor to treatment failure in many settings, potentially increasing the risk of refractory disease. Currently, there is a lack of early warning systems and standardized predictive models for RMPP, leading to delayed clinical interventions and affecting patient outcomes (8). In recent years, numerous studies have explored risk prediction models for pediatric RMPP, but further research is needed to determine the predictive ability and clinical utility of these models (9, 10).

Therefore, the aim of this study was to identify early serum biomarkers associated with RMPP and to construct an externally validated, multi-factor predictive model that integrates these biomarkers to distinguish RMPP from GMPP in children, while also characterizing key clinical and radiologic differences between the two groups.

## 2 Methods

### 2.1 Case selection

This retrospective study included 568 children diagnosed with *Mycoplasma pneumoniae* pneumonia (MPP) who were hospitalized in the Department of Pediatrics at Nanjing Jiangbei Hospital. The study protocol was reviewed and approved by the Ethics Committee of Nanjing Jiangbei Hospital. Given the retrospective design and use of de-identified data, the requirement for informed consent was waived; no potential risks or adverse consequences affected the patients.

All included patients were ≤14 years of age and presented with symptoms of lower respiratory tract infection, new pulmonary infiltrates on chest imaging, and laboratory evidence of MP infection. MPP diagnosis required compatible clinical/radiologic findings plus MP confirmation by PCR from respiratory specimens and/or positive MP-IgM serology obtained during hospitalization (Section 2.2.3).

RMPP was defined according to widely used pediatric diagnostic criteria reported in recent authoritative studies. Specifically, RMPP was diagnosed when children with confirmed MPP developed persistent fever ≥38.0°C and/or clinical deterioration accompanied by radiologic progression (such as enlarging consolidation, atelectasis, or new/worsening pleural effusion) despite ≥7 days of appropriate macrolide therapy (1).

Patients were excluded if they had: (1) other confirmed respiratory tract infections as the primary diagnosis (e.g., bacterial pneumonia unrelated to MP, viral bronchiolitis), (2) chronic lung disease (e.g., bronchopulmonary dysplasia, bronchiectasis), (3) congenital heart disease, (4) chronic liver or kidney disease, (5) rheumatic or autoimmune disease, (6) primary or secondary immunodeficiency, or (7) incomplete key clinical or laboratory data required for the predictive model.

Children with a primary diagnosis of typical bacterial pneumonia were excluded to ensure that all included patients had *Mycoplasma pneumoniae* pneumonia (MPP) as the main diagnosis. When additional bacterial pathogens were detected, clinicians determined whether these represented secondary co-infection or primary bacterial pneumonia. Only cases in which MPP remained the primary cause of illness were included.

### 2.2 Data collection

#### 2.2.1 Demographic and clinical information

Demographic and clinical information were retrospectively extracted from the electronic medical records, including age, sex, weight, history of allergic disease, and previous respiratory infections. Clinical variables included duration of symptoms before admission, duration of fever, presence of tachypnea, wheezing, chest tightness, chills, and extrapulmonary manifestations.

For each patient, we recorded the date of symptom onset, the date of hospital admission, and the date of initiation of macrolide therapy. From these, we calculated the interval from symptom onset to admission and from symptom onset to the start of macrolide therapy (days) and compared these timing variables between the GMPP and RMPP groups in the descriptive analyses.

Fever duration was defined as the number of days during which the axillary temperature exceeded 38.0°C at least once per day (11). Tachypnea was defined according to age-specific respiratory rate thresholds in national pediatric CAP guidelines (12). Information on prior outpatient therapy (including any macrolide use before admission), in-hospital antibiotic regimens, systemic corticosteroid use, and immunoglobulin therapy was also recorded.

#### 2.2.2 Laboratory data

##### 2.2.2.1 Timing of blood sampling

For all patients, peripheral venous blood samples for baseline laboratory testing were collected within 24 h of hospital admission and before initiation of systemic corticosteroids, intravenous immunoglobulin, or escalation to second-line antibiotics whenever possible. When patients had received macrolides prior to admission, baseline laboratory data still reflected the earliest available in-hospital blood draw. For the predictive analyses, only these baseline values were used. In patients with persistent fever or clinical deterioration, complete blood count (CBC) and C-reactive protein (CRP) were repeated every 2–3 days as clinically indicated, but repeat values were not used for model development.

##### 2.2.2.2 Routine hematologic and biochemical indices

CBC, including total leukocyte count and neutrophil percentage, was measured using an automated hematology analyzer (Sysmex XN-1000, Sysmex Corporation, Kobe, Japan). CRP, lactate dehydrogenase (LDH), and prealbumin (PAB) were measured on an automated chemistry analyzer (Cobas c701/c702, Roche Diagnostics, Mannheim, Germany) using standard immunoturbidimetric or enzymatic methods according to the manufacturer's instructions.

Serum immunoglobulin A (IgA), immunoglobulin G (IgG), immunoglobulin M (IgM), and immunoglobulin E (IgE), as well as complement components C3 and C4, were determined by immunoturbidimetry on the same analyzer platform. All assays were calibrated and quality-controlled according to the manufacturer's recommendations and the hospital laboratory's internal quality management program.

##### 2.2.2.3 Cytokines

Serum levels of interleukin-2 (IL-2), IL-4, IL-6, IL-10, tumor necrosis factor-α (TNF-α), and interferon-γ (IFN-γ) were quantified using a cytometric bead array (CBA Human Th1/Th2 Cytokine Kit II, BD Biosciences, San Diego, CA, USA). Samples were processed in batches; after incubation with capture beads and detection reagents according to the kit protocol, fluorescence signals were acquired on a FACSCanto II flow cytometer (BD Biosciences). Cytokine concentrations were calculated using BD FCAP Array or CBA analysis software based on standard curves generated for each cytokine.

##### 2.2.2.4 T-cell subsets and other lymphocyte parameters

Peripheral blood *T*-cell subsets were assessed by flow cytometry. Whole blood was stained with BD Multitest 6-Color TBNK reagent (BD Biosciences), which simultaneously identifies CD3+ *T* cells and CD3+ CD4+ (helper) and CD3+ CD8+ (cytotoxic) *T*-cell subsets, as well as B cells and NK cells. After red cell lysis and washing, samples were analyzed on a FACSCanto II cytometer, and lymphocyte subsets were quantified using the manufacturer's analysis templates. Absolute counts were derived from the analyzer-reported lymphocyte count and subset percentages.

Neutrophil percentage (NEU%) and absolute neutrophil counts were obtained from the automated hematology analyzer (Sysmex XN-1000) as part of the CBC.

#### 2.2.3 Respiratory specimen testing

Nasopharyngeal or throat swab specimens were collected within 24 h of admission by trained nurses using flocked swabs and placed immediately into viral transport medium. For some patients, nasopharyngeal aspirates were obtained instead of swabs, following standard procedures.

Specimens were first processed for routine bacterial culture when clinically indicated. Respiratory viruses (e.g., respiratory syncytial virus, influenza A/B, adenovirus, parainfluenza viruses) were screened using direct immunofluorescence assays on epithelial cells from the nasopharyngeal samples according to manufacturer instructions.

When bacterial pathogens were isolated from respiratory or blood cultures, the treating physicians evaluated whether these findings indicated secondary bacterial co-infection. Children in whom bacterial pneumonia was considered the primary illness were excluded. For all included patients, suspected secondary co-infection did not determine group assignment; classification into GMPP vs. RMPP was based solely on the clinical and radiologic response to macrolide therapy.

##### 2.2.3.1 MP serology

Serum *Mycoplasma pneumoniae*-specific IgM antibodies were measured using a commercial ELISA kit (*Mycoplasma pneumoniae* IgM ELISA, DaAn Gene Co., Ltd., Guangzhou, China) according to the manufacturer's instructions. Optical density was read on an automated microplate reader, and results were classified as positive, negative, or equivocal based on the cut-off values provided in the kit insert. In the clinical setting, either a positive IgM result or a significant rise in antibody titer on paired sera supported the diagnosis of acute MP infection, in conjunction with clinical and radiologic findings.

##### 2.2.3.2 MP DNA detection by PCR

MP DNA was detected using a quantitative real-time PCR assay with a commercial diagnostic kit targeting the 16S rRNA gene of *Mycoplasma pneumoniae* [Diagnostic Kit for *Mycoplasma pneumoniae* DNA (PCR-Fluorescence Probing), DaAn Gene Co., Ltd., Guangzhou, China] on a Rotor-Gene Q real-time PCR platform (Qiagen, Hilden, Germany). All steps, including nucleic acid extraction, reaction setup, and cycling conditions, followed the manufacturer's protocol. Negative and positive controls supplied in the kit were included in each run. A cycle threshold (Ct) value within the kit-specified positive range was interpreted as MP DNA positive. Because a validated commercial kit was used, primer and probe sequences were proprietary and are not reported here.

Genotypic testing for macrolide resistance (e.g., 23S rRNA point mutations) was not routinely performed during the study period and was therefore not available for analysis.

#### 2.2.4 Treatment and monitoring

Most patients received macrolide antibiotics (azithromycin or erythromycin) as first-line therapy, consistent with national consensus guidelines for the management of community-acquired pneumonia in children in China (12). Supportive treatments included antipyretics, oxygen therapy, bronchodilators, and fluid management as clinically indicated.

For patients who met criteria for RMPP (persistent fever, worsening clinical signs, or radiologic progression after ≥7 days of macrolide therapy), escalation strategies such as systemic corticosteroids and/or intravenous immunoglobulin were considered according to the treating physician's judgment and national recommendations. Throughout hospitalization, patients were monitored for changes in vital signs, respiratory status, radiographic findings, and laboratory parameters until clinical improvement and discharge.

Broad-spectrum antibiotics (e.g., β-lactam/β-lactamase inhibitor combinations or cephalosporins) were prescribed only when bacterial co-infection was suspected based on clinical deterioration, imaging findings, and/or microbiologic testing, at the discretion of the treating team. Pulmonary complications were monitored through clinical reassessment and repeat chest imaging (radiography, and CT or ultrasound when clinically indicated), focusing on progression to consolidation, atelectasis, pleural effusion, and other complications recorded in Table 3.

**Table 3:** Radiological features between the two groups.

Parameters	GMPP group (*n* = 438)	RMPP group (*n* = 130)	*χ* ^2^	*P*
Patients with large lesions, %	152 (34.70%)	93 (71.54%)	55.453	<0.001
Patients with pulmonary complications, %				
Pleural effusion	49 (11.19%)	63 (48.46%)	87.984	<0.001
Lobar atelectasis	49 (11.19%)	34 (26.15%)	17.997	<0.001
Pulmonary consolidation	18 (4.11%)	29 (22.31%)	43.740	<0.001
Pleural thickening	16 (3.65%)	15 (11.54%)	12.081	<0.001
Necrotizing pneumonia	2 (0.46%)	2 (1.54%)	0.487	0.485

Importantly, escalation therapies (broad-spectrum antibiotics, corticosteroids, IVIG) were not used as model predictors because they typically occur after baseline assessment and may introduce treatment leakage; therefore, the predictive model was intentionally restricted to baseline serum biomarkers measured within 24 h of admission.

The use of additional broad-spectrum antibiotics was not used to define RMPP. Group allocation (GMPP vs. RMPP) was determined exclusively by the predefined clinical and radiologic criteria under macrolide treatment.

### 2.3 Statistical analysis and model development

Data processing and analysis were performed using SPSS 29.0 statistical software (IBM Corp., Armonk, NY, USA) and R software (version X.X.X; R Foundation for Statistical Computing, Vienna, Austria). Categorical variables were summarized as counts and percentages and compared between the RMPP and GMPP groups using the *χ*^2^ test or Fisher's exact test, as appropriate. Continuous variables were assessed for normality and summarized as mean ± standard deviation (SD) or median (interquartile range, IQR); between-group comparisons used the independent-samples *t*-test or Mann–Whitney *U* test. A *p*-value < 0.05 was considered statistically significant. Given the large number of baseline clinical and laboratory comparisons, these hypotheses tests were considered exploratory; *p*-values were interpreted descriptively and no formal adjustment for multiple comparisons was applied. Hyperparameters were optimized using repeated 10-fold cross-validation (5 repeats) within the development dataset. Because the proportion of missingness was low (<5% for all predictors), no imputation strategy was applied.

Because the outcome prevalence was imbalanced (RMPP 130/568, 22.9%), we used outcome-stratified resampling so that each cross-validation fold preserved similar RMPP/GMPP proportions. Model discrimination was optimized using AUC, and we reported threshold-based metrics (sensitivity, specificity, PPV, and NPV) at the Youden-optimal probability cut-off to transparently reflect performance under this prevalence.

Given the limitations of single biomarker prediction, the clinical utility of the prediction model was enhanced by integrating multiple significant biomarkers. While individual serum markers, such as CRP, LDH, and IFN-γ, demonstrated moderate to good discriminatory abilities, their standalone predictive value was insufficient for clinical decision-making. As a result, a multi-factor prediction model was developed, incorporating these biomarkers alongside additional clinical and laboratory features to ensure a more comprehensive evaluation of the risk of RMPP. The random forest algorithm was employed to combine the most relevant predictors, optimizing model performance and increasing its clinical applicability.

#### 2.3.1 Handling of missing data

For descriptive analyses, all available data were used. For regression and model-building analyses, we adopted a complete-case approach: patients with missing values for any of the candidate predictors included in a given model were excluded from that specific analysis. The proportion of missingness was low and did not cluster around a particular variable, so no formal imputation was performed.

#### 2.3.2 Pre-processing of predictor variables

All laboratory variables were used on their original measurement scales. Because random forests are relatively robust to differences in variable scaling, we did not perform standardization or normalization before model fitting. Implausible extreme values were checked against the original medical record; when confirmed to be data entry errors, they were corrected; otherwise, observed extreme values were retained in the analysis.

#### 2.3.3 Logistic regression analyses

To identify serum biomarkers associated with RMPP, univariate logistic regression analyses were first performed for each candidate biomarker. Variables with *p* < 0.10 in univariate analyses and those considered clinically important were then entered into multivariable logistic regression models to identify independent predictors of RMPP. Logistic regression analyses were conducted in SPSS 29.0, and odds ratios (ORs) with 95% confidence intervals (CIs) were reported.

#### 2.3.4 Random forest model

Because no single biomarker is sufficient for reliable clinical prediction, we next developed a multi-factor predictive model that integrates the most informative serum markers. A random forest model was developed to construct a combined predictive model for RMPP using the serum biomarkers that were statistically and clinically significant. Model development and validation were carried out in R using the randomForest package for model fitting and the caret package for resampling and performance evaluation. The dataset from Nanjing Jiangbei Hospital served as the development cohort. Within this cohort, hyperparameters (number of trees, number of variables tried at each split, and minimum node size) were tuned using grid search with 10-fold cross-validation to optimize discrimination.

#### 2.3.5 Internal and external validation

Internal performance was evaluated in the development cohort by estimating the area under the receiver operating characteristic curve (AUC), along with 95% CIs, and by calculating sensitivity, specificity, positive predictive value (PPV), and negative predictive value (NPV) at the optimal cut-off selected by the Youden index. Calibration of the model was assessed using calibration plots and the Brier score.

Furthermore, 105 patients from an external institution who met the same inclusion and exclusion criteria and outcome definitions (RMPP vs. GMPP) were included as an external validation cohort. The external validation cohort consisted of consecutive MPP cases admitted to a different tertiary hospital in Jiangsu Province during a later time period, using identical inclusion and exclusion criteria. The same predictor variables were available and measured in the same way. In this external cohort, the final model trained in the development cohort was applied without re-fitting to generate predicted probabilities of RMPP. AUC (with 95% CI), sensitivity, specificity, PPV, NPV, and calibration metrics were calculated to confirm the robustness and generalizability of the model.

The final random forest model, including parameter settings and analysis code, is available from the corresponding author upon reasonable request.

## 3 Results

### 3.1 Clinical information of patients

In the comparison of clinical characteristics between the GMPP group (*n* = 438) and the RMPP group (*n* = 130), significant differences were observed in several parameters (Table 1). Patients in the RMPP group were significantly older than those in the GMPP group (*P* < 0.001). The prevalence of fever (*P* < 0.001), tachypnea (*P* < 0.001), chest tightness (*P* = 0.038), chill (*P* = 0.013), extra-pulmonary complications (*P* < 0.001), and decreased unilateral lung sound (*P* < 0.001) was higher in the RMPP group. In contrast, wheezing was less frequent among RMPP patients than GMPP patients (1.54% vs. 14.38%, *P* < 0.001). There were no significant differences in sex distribution, presence of cough, or rates between the two groups (all *P* > 0.05).

**Table 1:** Clinical characteristic between the two groups.

Parameters	GMPP group (*n* = 438)	RMPP group (*n* = 130)	t/*χ*^2^	*P*
Age, years	4.63 ± 1.92	6.84 ± 2.15	11.207	<0.001
Sex (male/female)	245 (55.94%)/193 (44.06%)	62 (47.69%)/68 (52.31%)	2.743	0.098
Clinical presentation, *n* (%)				
Fever	378 (86.30%)	130 (100%)	19.912	<0.001
Cough	438 (100%)	130 (100%)	None	1
Tachypnea	28 (6.39%)	31 (23.85%)	32.806	<0.001
Chest tightness	2 (0.46%)	4 (3.08%)	4.317	0.038
Wheezing	63 (14.38%)	2 (1.54%)	16.321	<0.001
Chill	9 (2.05%)	9 (6.15%)	6.237	0.013
Extra-pulmonary complications	57 (13.01%)	51 (39.23%)	44.746	<0.001
Physical examination, *n* (%)				
Rales	290 (66.21%)	77 (59.23%)	2.136	0.144
Decreased unilateral lung sound	54 (12.33%)	53 (40.77%)	53.034	<0.001
Length of fever, days	8.25 ± 2.10	14.26 ± 3.45	18.833	<0.001
Length of stay, days	7.61 ± 1.85	11.45 ± 2.62	15.585	<0.001
Management				
Length of antibiotic therapy days	9.07 ± 1.52	16.09 ± 2.51	30.274	<0.001
Oxygenotherapy, *n* (%)	24 (5.48%)	36 (27.69%)	52.355	<0.001
ICU, *n* (%)	0 (0.00%)	4 (3.08%)	9.529	0.002

ICU, intensive care unit.

The RMPP group also had a longer duration of fever, longer length of hospital stay, and longer course of antibiotic therapy compared with the GMPP group (all *P* < 0.001). The use of oxygenotherapy (*P* < 0.001) and admission to the intensive care unit (ICU) (*P* = 0.002) were more common in the RMPP group.

For timing variables, the median interval from symptom onset to hospital admission and from symptom onset to initiation of macrolide therapy did not differ significantly between GMPP and RMPP (both *P* > 0.05). Therefore, the more severe course observed in RMPP is unlikely to be explained solely by delayed presentation or delayed macrolide initiation.

### 3.2 Laboratory characteristics of patients

**Figure 1:**
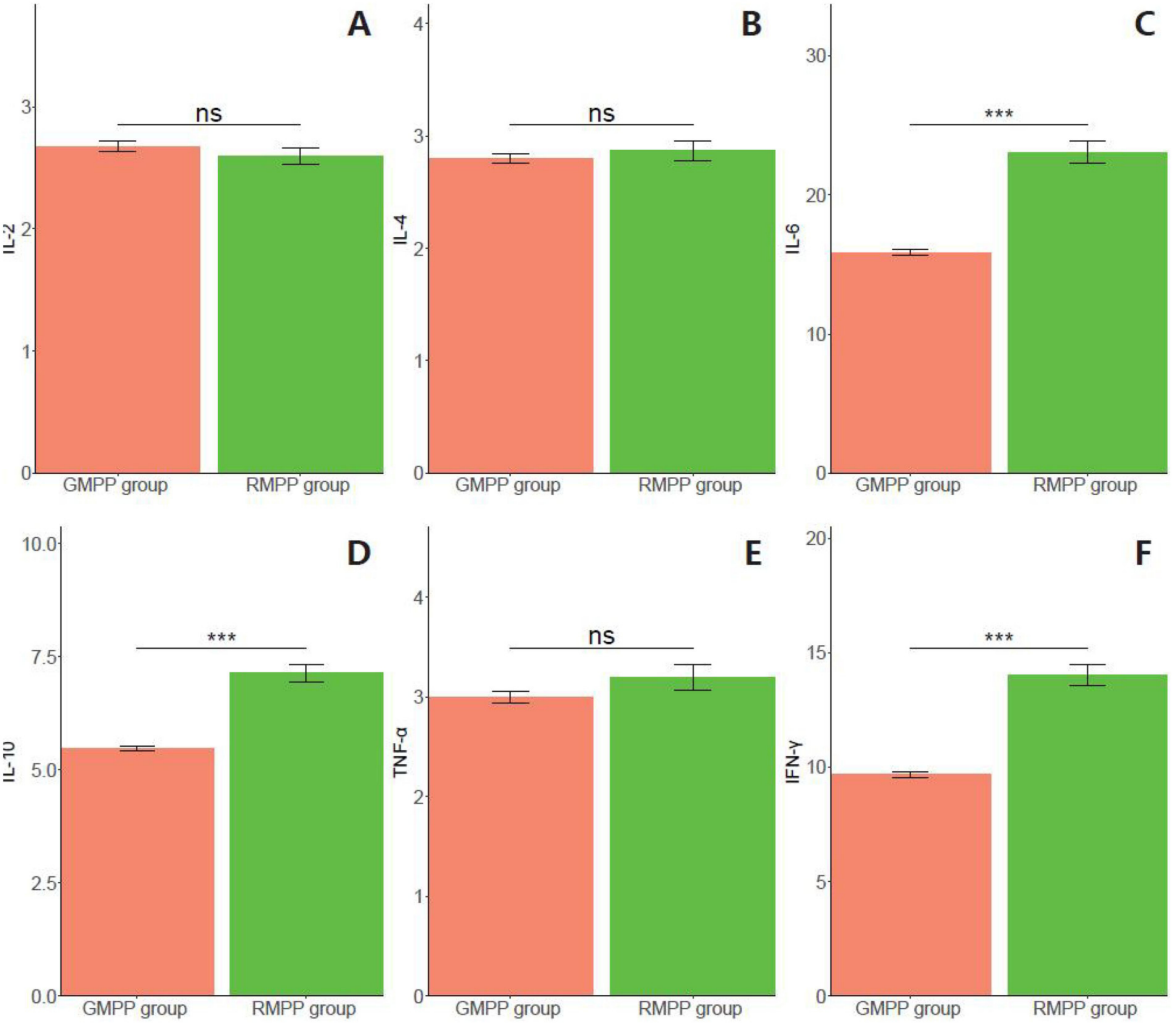
Cytokines between the two groups (pg/ml). **(A)** IL-2 (Interleukin 2); **(B)** IL-4 (Interleukin 4); **(C)** IL-6 (Interleukin 6); **(D)** (IL-10: Interleukin 10); **(E)** TNF-α (Tumor necrosis factor alpha); **(F)** IFN-γ (Interferon gamma). Ns, no statistically significant difference; **: *p* < 0.001.

The comparison of laboratory characteristics between the GMPP and RMPP groups revealed several notable differences (Table 2; Figure 1). Neutrophil percentage, CRP levels, and LDH levels were all significantly higher in the RMPP group than in the GMPP group (all *P* < 0.001), whereas PAB concentrations were significantly lower in RMPP (*P* < 0.001). Among immunoglobulins, IgA levels were significantly elevated in the RMPP group (*P* < 0.001), while IgG and IgM did not differ significantly between groups (both *P* > 0.05).

**Table 2:** Laboratory characteristics between the two groups.

Parameters	GMPP group (*n* = 438)	RMPP group (*n* = 130)	t	*P*
WBC (×10^9^ /L)	8.35 ± 3.18	7.74 ± 3.10	1.940	0.053
Neutrophil, %	57.23 ± 16.44	70.30 ± 10.78	10.635	<0.001
CRP (mg/L)	9.76 ± 2.63	15.13 ± 6.04	9.867	<0.001
LDH (IU/L)	378.33 ± 110.37	500.67 ± 183.00	7.241	<0.001
PAB (mg/dL)	12.50 ± 4.02	8.25 ± 4.01	10.605	<0.001
Total Ig (mg/dL)				
IgG	906.12 ± 98.93	896.28 ± 93.78	1.008	0.314
IgA	85.15 ± 20.29	101.17 ± 19.37	7.989	<0.001
IgM	154.62 ± 27.69	160.75 ± 32.74	1.940	0.054
Subpopulations of T lymphocytes, %				
CD3+	61.90 ± 11.24	63.10 ± 11.82	1.058	0.291
CD4+	33.84 ± 8.17	33.79 ± 9.53	0.049	0.961
CD8+	20.98 ± 6.60	22.67 ± 7.84	2.239	0.026

WBC, white blood cell; CRP, C-reactive protein; LDH, lactate dehydrogenase; PAB, prealbumin; Ig, immunoglobulin.

With respect to lymphocyte subsets, there were no significant differences in CD3+ or CD4+ *T*-cell percentages, but CD8+ *T*-cell percentage was modestly higher in the RMPP group (*P* = 0.026). Given the number of comparisons performed, this CD8+ difference should be interpreted cautiously as an exploratory finding.

Cytokine profiles showed a clear pattern of enhanced inflammatory activation in RMPP. IL-6, IL-10, and IFN-γ levels were all significantly higher in the RMPP group (all *P* < 0.001), whereas IL-2, IL-4, and TNF-α did not differ significantly between groups (all *P* > 0.05) (Figure 1). Taken together, these results suggest that RMPP is characterized by a more pronounced systemic inflammatory and immune response than GMPP. However, because multiple laboratory markers were evaluated, these group differences should be considered hypothesis-generating and interpreted in the context of the exploratory nature of the comparisons.

### 3.3 Radiological features of patients

In our study comparing radiological features between the GMPP group and the RMPP group, significant differences were observed in several parameters indicative of more extensive lung involvement in RMPP (Table 3). A significantly higher proportion of patients in the RMPP group had large lesions on chest imaging compared with the GMPP group (71.54% vs. 34.70%, *P* < 0.001). Radiographic pulmonary complications were also more frequent among RMPP patients: pleural effusion (48.46% vs. 11.19%, *P* < 0.001), lobar atelectasis (26.15% vs. 11.19%, *P* < 0.001), pulmonary consolidation (22.31% vs. 4.11%, *P* < 0.001), and pleural thickening (11.54% vs. 3.65%, *P* < 0.001). In contrast, there was no significant difference in the occurrence of necrotizing pneumonia between groups (*P* = 0.485).

Overall, these findings indicate that children with RMPP are more likely to develop extensive and complicated radiologic manifestations than those with GMPP, consistent with a more severe disease course. As with the clinical comparisons, these radiologic differences are based on multiple descriptive tests and should be interpreted as exploratory, but they support the notion that RMPP is associated with more aggressive pulmonary involvement.

### 3.4 Regression analysis predicting serum biomarkers for RMPP

Univariate logistic regression was used as an initial screening step to identify candidate serum biomarkers associated with RMPP. Biomarkers meeting the screening criterion (*p* < 0.10) and those with clinical relevance were then entered into a multivariable logistic regression model to identify independent predictors (Table 4).

**Table 4:** Multivariate logistic regression analysis predicting serum biomarkers for RMPP.

Parameters	Coefficient	Std_Error	Wald	*P*	OR	95%CI
Neutrophil, %	0.073	0.015	4.794	<0.001	1.076	1.044–1.109
CRP (mg/L)	0.340	0.057	5.967	<0.001	1.405	1.257–1.572
LDH (IU/L)	0.005	0.002	3.190	0.001	1.005	1.002–1.008
PAB (mg/dL)	−0.315	0.062	−5.060	<0.001	0.730	0.646–0.824
IgA (pg/ml)	0.045	0.011	4.131	<0.001	1.046	1.024–1.069
IL-6 (pg/ml)	0.156	0.034	4.545	<0.001	1.169	1.093–1.250
IL-10 (pg/ml)	0.457	0.132	3.456	<0.001	1.579	1.219–2.046
IFN-γ (pg/ml)	0.284	0.061	4.648	<0.001	1.329	1.179–1.498

CRP, C-reactive protein; LDH, lactate dehydrogenase; PAB, prealbumin; IgA, immunoglobulin A; IL-6, interleukin 6; IL-10, interleukin 10.

In the multivariable logistic regression model, which included variables with *P* < 0.10 in univariate analyses and those of clinical importance, several biomarkers remained independently associated with RMPP (Table 4). Neutrophil percentage (OR = 1.076 per 1% increase, *P* < 0.001), CRP (OR = 1.405 per 1 mg/L increase, *P* < 0.001), LDH (OR = 1.005 per 1 IU/L increase, *P* = 0.001), IgA (OR = 1.046 per 1 mg/dL increase, *P* < 0.001), IL-6 (OR = 1.169 per 1 pg/mL increase, *P* < 0.001), IL-10 (OR = 1.579 per 1 pg/mL increase, *P* < 0.001), and IFN-γ (OR = 1.329 per 1 pg/mL increase, *P* < 0.001) were all positively associated with RMPP, whereas PAB remained negatively associated (OR = 0.730 per 1 mg/dL increase, *P* < 0.001). Although the OR for LDH appears close to 1.0 per unit because it is expressed per 1 IU/L, over a more clinically meaningful range the effect is substantial: a 100 IU/L increase in LDH corresponds to an OR of approximately exp(0.005 × 100) = 1.65 for RMPP. Together, these results indicate that a composite pattern of elevated inflammatory and immune activation markers and reduced PAB is strongly and independently associated with RMPP, supporting their use as candidate predictors in the combined random forest model. Given the small unit scale, the effect of LDH is more clinically meaningful over larger increments; for example, a 100 IU/L rise corresponds to an OR 1.65.

### 3.5 Predictive values of the serum biomarker

The predictive performance of individual serum biomarkers for RMPP was evaluated using ROC analysis (Table 5). Neutrophil percentage, CRP, LDH, PAB, IgA, IL-6, IL-10, and IFN-γ all showed moderate to good discriminatory ability, with AUCs ranging from 0.714 to 0.790. Among these, CRP (AUC = 0.790) and IFN-γ (AUC = 0.771) had the highest AUCs, followed closely by PAB (AUC = 0.776) and IL-6 (AUC = 0.761).

**Table 5:** Predictive values of the serum biomarker in patients with RMPP.

Parameters	Cutoff value	Sensitivity	Specificity	AUC	PPV	NPV
Neutrophil, %	57.29	0.923	0.489	0.740 (95% CI: 0.687–0.793)	**0.349**	**0.955**
CRP (mg/L)	13.735	0.577	0.945	0.790 (95% CI: 0.741–0.839)	**0.757**	**0.883**
LDH (IU/L)	491.995	0.523	0.861	0.714 (95% CI: 0.660–0.768)	**0.528**	**0.859**
PAB (mg/dL)	11.255	0.815	0.616	0.776 (95% CI: 0.726–0.826)	**0.386**	**0.918**
IgA (mg/dL)	87.365	0.792	0.55	0.717 (95% CI: 0.663–0.771)	**0.343**	**0.899**
IL-6 (pg/ml)	20.695	0.631	0.833	0.761 (95% CI: 0.710–0.812)	**0.529**	**0.884**
IL-10 (pg/ml)	6.7	0.600	0.847	0.736 (95% CI: 0.683–0.789)	**0.538**	**0.877**
IFN-γ (pg/ml)	13.78	0.569	0.945	0.771 (95% CI: 0.720–0.822)	**0.754**	**0.881**

CRP, C-reactive protein; LDH, lactate dehydrogenase; PAB, prealbumin; IgA, immunoglobulin A; IL-6, interleukin 6; IL-10, interleukin 10.

Bold values indicate statistical significance at *P* < 0.05.

For each biomarker, optimal cut-off values were selected by maximizing Youden's index, and the corresponding sensitivities and specificities are summarized in Table 5. For example, at their respective cut-offs, CRP and IFN-γ achieved high specificities (0.945 for CRP and 0.945 for IFN-γ), whereas neutrophil percentage and PAB provided higher sensitivities (0.923 and 0.815, respectively). AUC estimates together with their 95% confidence intervals (Table 5) indicate that all eight biomarkers discriminate RMPP from GMPP better than chance, supporting their use as candidate predictors in the combined model rather than stand-alone diagnostic tests.

Accordingly, these single-marker ROC analyses were used mainly to compare relative discriminatory ability and to select candidate predictors, whereas the primary focus of this study is the combined multi-marker model described in Sections 3.6–3.8.

### 3.6 Establishment of combined predictive model

Given the limited clinical value of single biomarkers in isolation, we next constructed a multi-factor predictive model that integrates the eight independently associated serum markers.

**Figure 2:**
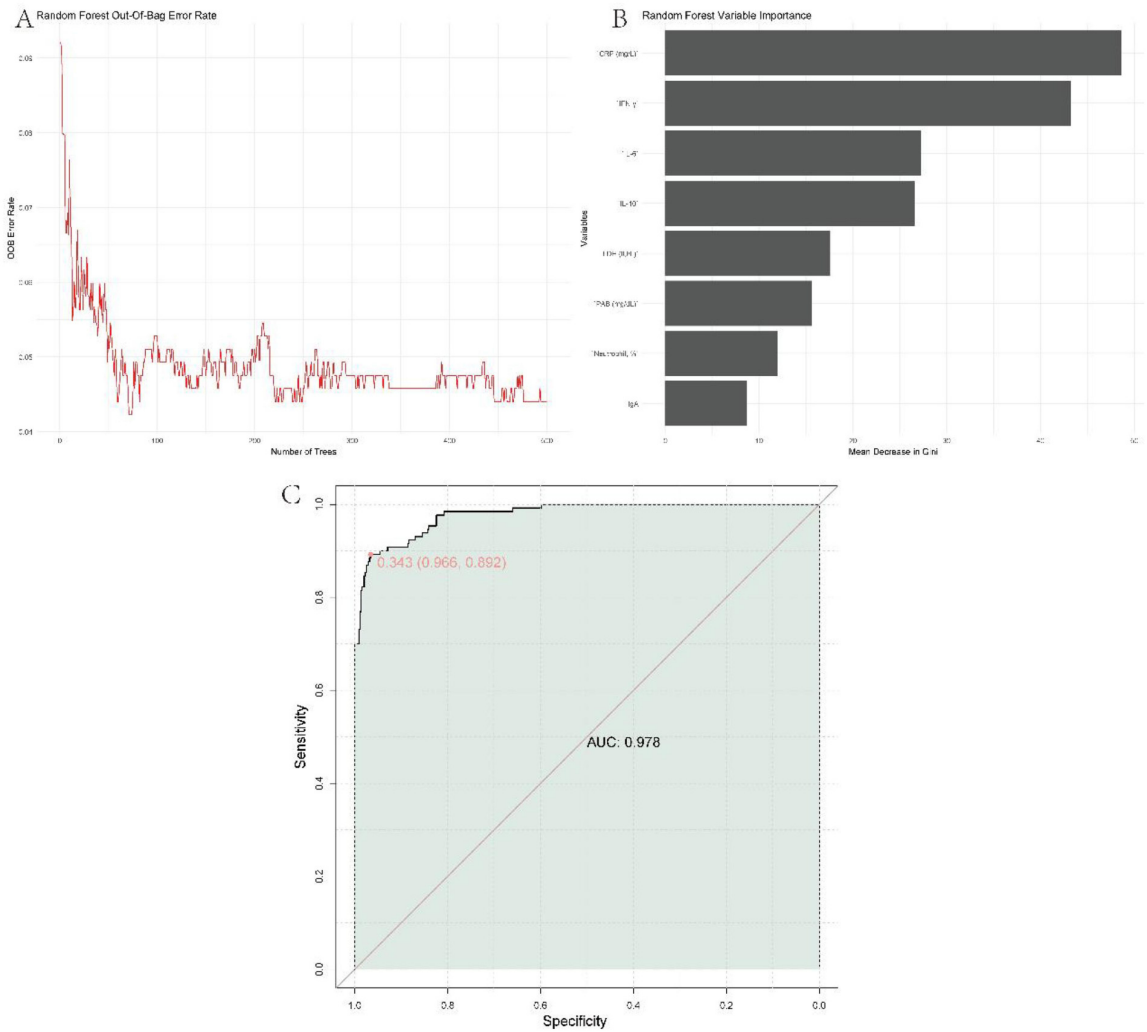
Establishment of a combined predictive model. **(A)** Out-of-bag error rate plot; **(B)** random forest variable importance; **(C)** ROC curve.

A combined predictive model for RMPP was constructed using a random forest algorithm incorporating the eight independently associated serum biomarkers (neutrophil percentage, CRP, LDH, PAB, IgA, IL-6, IL-10, and IFN-γ). In the development cohort, this model showed excellent discrimination, with an AUC of 0.978 [95% CI: (insert from ROC analysis); Figure 2C].

Variable-importance analysis (Figure 2B) indicated that CRP and IFN-*γ* contributed most strongly to the model, followed by IL-6, IL-10, LDH, PAB, neutrophil percentage, and IgA. The out-of-bag error rate stabilized after approximately 50 trees and remained low thereafter (Figure 2A), supporting model stability.

Using the optimal probability cut-off of 0.343 (chosen by maximizing Youden's index), the model achieved a sensitivity of 0.892 and a specificity of 0.966 for identifying RMPP. At this threshold, the positive predictive value and negative predictive value were approximately 0.89 and 0.97, respectively, reflecting both a high probability that predicted RMPP truly had RMPP and a very low probability of RMPP among those classified as low-risk. Calibration was good, with a low Brier score and close agreement between predicted and observed RMPP probabilities across risk strata, implying that predicted risks are well aligned with actual outcomes in the development cohort. For comparison, a traditional multivariable logistic regression model using the same predictors achieved an AUC of 0.854 in the development cohort, lower than the random forest model, supporting the superior discriminative performance of the non-linear machine-learning approach.

While individual biomarkers, such as CRP, LDH, and IFN-γ exhibited moderate to good discriminatory power (AUCs ranging from 0.714 to 0.790), their predictive value was limited when used alone. To overcome this, a combined multi-factor model was developed, incorporating eight independent serum biomarkers (neutrophil percentage, CRP, LDH, PAB, IgA, IL-6, IL-10, and IFN-γ) to enhance predictive accuracy. This multi-factor approach significantly improved the model's performance, with an AUC of 0.978 (95% CI: 0.960–0.995), remarkably surpassing the discriminatory ability of single markers. The model demonstrated excellent calibration and robust external validation, further supporting its potential for clinical application in identifying patients at risk for RMPP.

Although the Youden-based threshold provides a balanced operating point, the probability cut-off can be tuned to prioritize sensitivity (minimizing missed RMPP cases) in settings where early escalation is desired, or to prioritize specificity where avoiding overtreatment is the priority.

### 3.7 External validation of the predictive model

For the external validation of the predictive model, 105 children with MPP from an independent institution were included, of whom 81 had GMPP and 24 had RMPP (Table 6). Similar to the development cohort, age was significantly higher in the RMPP group than in the GMPP group (*P* < 0.001), whereas sex distribution did not differ between groups (*P* = 0.402).

**Table 6:** Parameters between the two groups.

Parameters	GMPP group (*n* = 81)	RMPP group (*n* = 24)	t/χ^2^	*P*
Age, years	4.55 ± 1.86	6.73 ± 2.06	4.922	<0.001
Sex (male/female)	45 (55.56%)/36 (44.44%)	11 (45.83%)/13 (54.17%)	0.703	0.402
Clinical presentation, *n* (%)				
Fever	65 (80.25%)	24 (100%)	4.168	0.041
Cough	81 (100%)	24 (100%)	None	1
Tachypnea	5 (6.17%)	6 (25.00%)	5.134	0.023
Chest tightness	1 (1.23%)	5 (20.83%)	9.812	0.002
Wheezing	21 (25.93%)	1 (4.17%)	5.293	0.021
Chill	2 (2.47%)	5 (20.83%)	7.300	0.007
Extra-pulmonary complications	11 (13.58%)	9 (37.50%)	5.406	0.020
Physical examination, *n* (%)				
Rales	53 (65.43%)	14 (58.33%)	0.404	0.525
Decreased unilateral lung sound	10 (12.35%)	10 (41.67%)	8.509	0.004
Length of fever, days	8.36 ± 2.09	14.37 ± 3.42	8.167	<0.001
Length of stay, days	7.54 ± 1.95	11.38 ± 2.52	7.912	<0.001
Management				
Length of antibiotic therapy days	9.14 ± 1.59	16.16 ± 2.58	12.633	<0.001
Oxygenotherapy, *n* (%)	5 (6.17%)	6 (25.00%)	5.134	0.023
ICU, *n* (%)	0 (0.00%)	4 (16.67%)	9.855	0.002
Patients with large lesions, %	28 (34.57%)	17 (70.83%)	9.943	0.002
Patients with pulmonary complications, %				
Pleural effusion	9 (11.11%)	11 (45.83%)	12.312	<0.001
Lobar atelectasis	8 (9.88%)	7 (29.17%)	4.161	0.041
Pulmonary consolidation	4 (4.94%)	5 (20.83%)	4.113	0.043
Pleural thickening	3 (3.70%)	5 (20.83%)	5.476	0.019
Necrotizing pneumonia	0 (0.00%)	0 (0.00%)	None	1
Neutrophil, %	59.30 ± 15.39	73.46 ± 10.21	5.253	<0.001
CRP (mg/L)	10.52 ± 2.82	15.21 ± 5.15	4.274	<0.001
LDH (IU/L)	381.66 ± 110.45	502.12 ± 183.27	3.060	0.005
PAB (mg/dL)	13.12 ± 4.21	9.18 ± 2.35	5.875	<0.001
IgA (mg/dL)	86.34 ± 19.27	108.25 ± 20.22	4.839	<0.001
CD8+, %	20.31 ± 6.59	23.52 ± 7.83	2.005	0.048
IL-6 (pg/ml)	13.15 ± 2.94	20.15 ± 10.33	3.277	0.003
IL-10 (pg/ml)	5.45 ± 1.13	7.09 ± 2.86	2.749	0.011
IFN-γ(pg/ml)	8.37 ± 2.70	12.60 ± 7.39	2.749	0.011

ICU, intensive care unit; CRP, C-reactive protein; LDH, lactate dehydrogenase; PAB, prealbumin; IgA, immunoglobulin A; IL-6, interleukin 6; IL-10, interleukin 10; IFN-γ, interferon gamma.

RMPP cases more frequently presented with fever (*P* = 0.041), tachypnea (*P* = 0.023), chest tightness (*P* = 0.002), chill (*P* = 0.007), extra-pulmonary complications (*P* = 0.020), and decreased unilateral lung sound (*P* = 0.004). Length of fever, length of hospital stay, and duration of antibiotic therapy were all significantly longer in the RMPP group (all *P* < 0.001). Oxygenotherapy and ICU admission were also more common among RMPP patients (*P* = 0.023 and *P* = 0.002, respectively).

Radiologically, patients with RMPP were more likely to have large lesions and pulmonary complications, including pleural effusion, lobar atelectasis, pulmonary consolidation, and pleural thickening (all *P* ≤ 0.043), mirroring the patterns observed in the development cohort. In addition, several serum biomarkers, neutrophil percentage, CRP, LDH, PAB, IgA, CD8+ percentage, IL-6, IL-10, and IFN-γ remained significantly different between GMPP and RMPP (all *P* ≤ 0.048). These external findings support the robustness of the clinical and laboratory differences associated with RMPP, although they are based on multiple descriptive comparisons and should be interpreted as exploratory.

The external validation cohort also demonstrated the clinical utility of the multi-factor model. In this cohort, the combined model achieved an AUC of 0.942 (95% CI: 0.915–0.968), confirming the model's robust performance and generalizability across different patient populations. By integrating multiple biomarkers, the model maintained high sensitivity (0.892) and specificity (0.966), indicating its capacity to accurately identify patients with RMPP and those at low risk, thereby improving clinical decision-making compared to individual biomarkers alone.

### 3.8 External validation ROC

**Figure 3:**
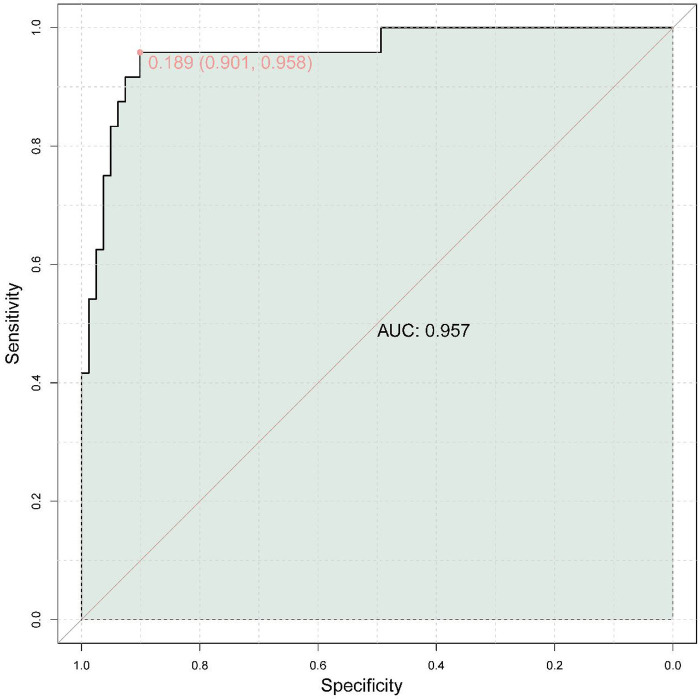
External validation ROC curve.

The performance of the random forest model in the external validation cohort is shown in Figure 3. The model maintained excellent discrimination, with an AUC of 0.957 (95% CI: 0.901–0.958). Using the optimal probability cut-off of 0.189, selected by maximizing Youden's index in the external dataset, the model achieved a sensitivity of 0.958 and a specificity of 0.901 for identifying RMPP. At this threshold, the positive predictive value was approximately 0.74 and the negative predictive value was approximately 0.99, indicating that most patients predicted to have RMPP truly had refractory disease, and that RMPP was very unlikely among those classified as low-risk. Calibration in the external cohort was also good, with a low Brier score and close agreement between predicted and observed RMPP probabilities across risk strata (calibration plot not shown). Overall, these results indicate that the combined biomarker model retains strong discriminative ability and good classification performance when applied to an independent population, supporting its potential clinical utility for early risk stratification of children with MPP.

Taken together, the integrated multi-marker model provides substantially better discrimination for RMPP than any individual laboratory parameter, and its performance is robust across independent cohorts.

## 4 Discussion

The present study aimed to identify early predictive serum biomarkers and construct a combined model for distinguishing RMPP from GMPP in children. We found that children with RMPP consistently exhibited more severe clinical manifestations, more extensive radiologic abnormalities, and a distinct pattern of inflammatory and immune biomarkers compared with those with GMPP. By integrating eight serum markers into a random forest model, we achieved excellent discrimination between RMPP and GMPP in both the development and external validation cohorts, suggesting that a multimarker approach may be useful for early risk stratification in clinical practice (13).

Clinically, RMPP patients in our cohort were older, had longer fever duration, more frequent extra-pulmonary manifestations, longer hospital stays, and higher rates of oxygenotherapy and ICU admission than GMPP patients. These findings are consistent with previous reports indicating that older children with MPP are more prone to severe or refractory courses, potentially reflecting age-related differences in host immune responses, including heightened Th1/Th2 cytokine activity (14, 15). Importantly, the intervals from symptom onset to hospital admission and from symptom onset to initiation of macrolide therapy did not differ significantly between GMPP and RMPP, suggesting that the more severe clinical course in RMPP is unlikely to be explained solely by delayed presentation or delayed initiation of macrolides. Instead, persistent inflammation and immune dysregulation are more plausible contributors to refractoriness (16).

Radiologically, RMPP was characterized by a higher prevalence of large lesions, pleural effusion, lobar atelectasis, pulmonary consolidation, and pleural thickening, whereas necrotizing pneumonia remained uncommon in both groups. These findings are in line with earlier studies linking RMPP to more extensive lung involvement and complicated imaging patterns (17, 18). In contrast, wheezing was less frequent in RMPP than GMPP, which may reflect differences in airway involvement vs. parenchymal disease. Reduced unilateral breath sounds were more common in RMPP, consistent with larger or more localized areas of consolidation or atelectasis. Together, these patterns support the concept that RMPP represents a more aggressive phenotype of MPP with greater structural lung injury.

Our biomarker analyses provide further insight into the pathophysiology of RMPP. Mechanistically, the selected biomarker panel reflects convergent inflammatory and immune pathways implicated in RMPP. IL-6 is a key upstream mediator of acute-phase responses and plausibly contributes to elevated CRP, while LDH reflects tissue injury and cellular damage; together, these markers align with reports that excessive host immune responses and inflammatory cascades are central to RMPP pathogenesis (1). In parallel, elevated IL-10 and IFN-γ suggest dysregulated immunomodulation (mixed anti-inflammatory feedback and Th1-skewed activation), which has been reported as clinically informative for distinguishing refractory from general MPP in children (19). These biologically linked signals support our use of a combined panel rather than reliance on any single marker.

Higher levels of CRP, LDH, neutrophil percentage, IgA, IL-6, IL-10, and IFN-γ, along with lower PAB levels, were independently associated with RMPP. LDH is a marker of cellular injury and tissue hypoxia; in our multivariable model, a seemingly small unit-based odds ratio for LDH (per 1 IU/L) corresponded to a substantial increase in RMPP risk over a clinically relevant range (e.g., a 100 IU/L increase) (1). CRP reflects the acute-phase response and has been repeatedly linked to severe or refractory MPP, likely capturing both pathogen burden and downstream inflammatory activation (20). Elevated IL-6, IL-10, and IFN-γ are consistent with a highly activated cytokine milieu and imbalance between pro- and anti-inflammatory pathways described in prior RMPP studies (15, 21). Neutrophilia and increased CD8+ *T*-cell activity may contribute to tissue damage through cytotoxic mechanisms and neutrophil-mediated injury (21, 22). Conversely, lower PAB levels are compatible with a catabolic, inflammatory state and have been associated with prolonged hospitalization and poorer recovery in pediatric infections (23). Elevated IgA may reflect enhanced mucosal immune activation and could contribute to inflammation via complement-mediated pathways (24). Although our observational design cannot establish causality, these biomarkers collectively delineate an exaggerated inflammatory and immune-activation profile in RMPP.

By integrating these markers, the random forest model achieved markedly better discrimination than any single biomarker alone. In the development cohort, the combined model showed an AUC of 0.978 with high sensitivity and specificity at the chosen cut-off, and excellent negative predictive value, suggesting potential clinical utility for early identification of children at high risk for RMPP. Importantly, performance remained strong in an independent external cohort (AUC 0.957), indicating that the model is robust and not limited to a single population (25, 26). Compared with traditional approaches relying on single laboratory indicators (e.g., CRP, LDH) or purely clinical criteria, our multimarker model may reduce false negatives and help clinicians identify high-risk patients earlier, when timely escalation to adjunctive therapies such as corticosteroids or immunoglobulin could be most effective (24, 26). Nevertheless, the model is intended to support, rather than replace, clinical judgment, and its cut-offs and performance will need confirmation in additional settings.

Compared with prior RMPP prediction approaches, our model showed strong discrimination (AUC 0.978; external AUC 0.957). For example, earlier work evaluating single or limited marker combinations reported AUCs typically in the moderate-to-high range (e.g., soluble fibrin, D-dimer, and CRP indices with AUCs around 0.81–0.90) (27, 28). More recently, machine-learning and score-based models have reported development AUCs around 0.91–0.93 with variable validation performance depending on cohort differences and feature sets (28). Our study extends this literature by emphasizing a compact baseline serum biomarker panel with clearly reported threshold metrics and independent external validation, supporting potential early risk stratification.

Although several individual markers such as CRP, IFN-γ, PAB, and IL-6 showed moderate-to-good discrimination, their stand-alone predictive performance is still insufficient for confident early risk stratification in routine practice. Our findings support the concept that combining multiple, biologically related markers of inflammation, immune activation, and nutritional status yields much stronger discriminative ability. The random forest model integrating eight serum biomarkers achieved an AUC of 0.978 in the development cohort and 0.957 in external validation, clearly outperforming any single biomarker and providing greater clinical application value than single-factor approaches.

Several limitations should be considered when interpreting our findings. First, this was a retrospective study from two institutions, and residual confounding and selection bias cannot be excluded. Although we adjusted for multiple biomarkers in multivariable analyses and used robust machine-learning methods, unmeasured clinical factors may still influence both biomarker levels and the risk of RMPP. Although we used stratified resampling and external validation, the class imbalance (RMPP 23%) may still affect threshold-dependent metrics, particularly PPV, and performance should be re-confirmed in larger multi-center cohorts.

Second, baseline clinical and laboratory differences between groups were assessed using numerous hypothesis tests; these comparisons were exploratory, and we did not formally adjust for multiple testing, so some statistically significant findings may be due to chance. Third, genotypic testing for macrolide resistance (e.g., 23S rRNA mutations) was not routinely performed during the study period, preventing us from directly evaluating the contribution of macrolide-resistant *M. pneumoniae* to RMPP in our cohort. Fourth, we focused on baseline biomarker levels obtained at admission and did not analyze dynamic changes over the course of treatment, which may carry additional prognostic information. Fifth, although the model performed well in an external validation cohort, that cohort was relatively small and drawn from a similar regional context, which may limit generalizability to other geographic settings, healthcare systems, or populations with different epidemiologic profiles (1). Macrolide resistance testing (e.g., 23S rRNA mutations) was not performed in this cohort, which may influence severity classification; this remains an important area for future work.

Although primary bacterial pneumonia cases were excluded, we did not systematically quantify secondary bacterial co-infection, and detailed microbiologic confirmation was not available for all patients. Therefore, the independent contribution of bacterial co-infection to RMPP cannot be fully separated in this cohort.

Future prospective studies with larger, more diverse cohorts are needed to validate and refine this model, incorporate dynamic biomarker trajectories, and integrate other data sources such as radiomics, proteomics, or host genetic polymorphisms relevant to cytokine signaling to further improve prediction and explore mechanistic pathways. Studies should also evaluate whether combining baseline biomarkers with standardized radiologic features (e.g., consolidation extent, pleural effusion, atelectasis) improves calibration and clinical utility.

Despite these limitations, our study provides a reproducible, externally validated serum-biomarker model that may help clinicians identify children with MPP who are at high risk for refractory disease and may benefit from closer monitoring and early consideration of adjunctive therapies.

## 5 Conclusion

This study establishes a biomarker panel and predictive model that effectively predicts RMPP risk in children by integrating inflammatory, immune, and nutritional parameters. The analysis of clinical, laboratory, and radiological data not only enhances diagnostic precision but also illuminates the complex pathophysiology of refractory mycoplasma infections. Clinically, early identification of high-risk patients using this model could guide personalized therapies, reduce complications, and improve outcomes.

## Data Availability

The original contributions presented in the study are included in the article/Supplementary Material, further inquiries can be directed to the corresponding author.
